# Firms’ participation in the Swiss COVID-19 loan programme

**DOI:** 10.1186/s41937-021-00070-4

**Published:** 2021-05-04

**Authors:** Lucas Marc Fuhrer, Marc-Antoine Ramelet, Jörn Tenhofen

**Affiliations:** Swiss National Bank, Börsenstrasse 15, Zürich, 8022 Switzerland

**Keywords:** COVID-19, Loan programme, Guarantees, Firm behaviour, D22, H81

## Abstract

This paper analyses the determinants of firm participation in the Swiss COVID-19 loan programme, which aims to bridge firms’ liquidity shortfalls that have resulted from the pandemic. State-guaranteed COVID-19 loans are widely used by Swiss firms, with 20% of all firms participating, resulting in a sizeable programme of 2.4% of GDP. We use a comprehensive dataset to study the determinants of firm participation. Our results can be summarised as follows. First, participation was largely driven by the exposure of a firm to lockdown restrictions and to the intensity of the virus in the specific region. Second, we show that firms associated with lower liquidity ratios had a significantly higher probability of participating in the programme. Third, we find no clear evidence that firm indebtedness affected participation in the programme and no evidence that pre-existing potential “zombie firms” participated more strongly in the loan programme. Fourth, we show that the programme reached younger and smaller firms, which could be financially more vulnerable as they are less likely to obtain outside finance during a crisis. Overall, we conclude that given its objective, the programme appears to be successful.

## Introduction

Aside from its impact on public health, the COVID-19 pandemic caused a major economic shock. Governments reacted with a series of large-scale economic measures, ranging from short-time work schemes to credit support facilities. In Switzerland, the COVID-19 emergency loan programme was one of the key measures used to address the economic fallout of the pandemic. The Federal Council announced the programme on 25 March 2020 and stated the following objective: “Last Friday, 20 March 2020, the Federal Council presented a comprehensive package of measures to cushion the economic impact of the coronavirus pandemic. Bridging credit facilities should provide companies with sufficient liquidity to cover their current overheads despite turnover reductions associated with the new coronavirus.”

This paper studies the key determinants of firm participation in the COVID-19 loan programme. The aim of our analysis is to assess whether the loan programme can be considered successful given the objective stated by the government. Additionally, we evaluate whether the programme comes with potential negative side effects. Understanding why firms chose to participate in the programme is important for at least two reasons. First, the success of the programme can be evaluated. Second, lessons can be learned for potential future loan programmes.

Participation in the COVID-19 loan programme was sizeable, as 20% of all firms participated in this programme comprising a guaranteed loan volume of 2.4% of annual GDP. Participation is even more sizeable when considering the fact that approximately 60% of all small and medium-sized enterprises (SMEs) in Switzerland were debt-free prior to the crisis. The COVID-19 loan programme enabled firms to receive a government-guaranteed bank loan quickly (usually within one business day) and with a minimum of bureaucracy. Firms could easily obtain the loan, and the requirements were minimal. As loans are guaranteed by the government and banks can refinance the loans at the central bank, loan supply was perfectly elastic. Because of this, whether a firm took a COVID-19 loan purely reflects loan demand. Loan conditions were also favourable and included a 100% guarantee and an attractive interest rate of 0% in the first year for small loans, as well as a rather long loan maturity of at least 5 years.^1^

We analyse firm participation in the COVID-19 programme by estimating a binary response model.^2^ Our analysis focuses on the following questions: first, we evaluate whether a firm’s exposure to lockdown restrictions and to the virus intensity in the specific region can explain its participation in the loan programme. Second, we assess whether firms associated with lower liquidity ratios had a higher participation rate. Likewise, we analyse whether participation is higher for more indebted firms and whether it is particularly more so for firms in a pre-existing potentially precarious financial situation, i.e. firms with a relatively low profitability and high indebtedness before the pandemic hit (“zombie firms”). Finally, we document whether the loan programme reached potentially more vulnerable firms, such as younger and smaller companies. To address these questions, we build a comprehensive dataset combining various data sources. In particular, we match the complete set of firms in Switzerland from the register of commerce (BUR database) to the list of firms participating in the loan programme (JANUS database).

Our findings can be summarised as follows. First, we show that participation in the loan programme is positively related to the exposure of a firm’s activity to lockdown restrictions as well as to the regional virus intensity, which we use as a proxy for households becoming more cautious. Second, we show that firms with an ex ante weaker liquidity position had a higher probability to participate in the programme. Importantly, these effects are economically meaningful; we can explain a wide range of firm participation rates. Hence, we find supporting evidence for the loan programme’s success in reaching its objective. Third, we find no clear evidence that firm indebtedness affected participation and no evidence that participation was higher for firms with an ex ante relatively low profitability and high indebtedness, i.e. what we identify as zombie firms.^3^ Fourth, we show that the programme reached younger and smaller firms. Hence, the loan programme reached firms for which access to outside finance is typically more challenging—particularly during a crisis. Overall, our results are robust to different specifications and rely on several measures that exploit variation across sectors, regions and firm sizes.^4^

Our contribution to the literature is twofold. First, our paper contributes to the growing literature that studies the COVID-19 loan programme in Switzerland. For instance, firm participation in the programme is analysed by Brülhart, Lalive, Lehmann, and Siegenthaler (2020) and Zoller-Rydzek and Keller (2020). Our paper complements these studies, which are based on surveys, by instead using a comprehensive dataset combining various data sources. Moreover, we use what we believe to be exogenous measures of lockdown restrictions at a relatively granular level. Additionally, we explicitly account for firms’ liquidity position, which seems to be an economically important driver for participation in the loan programme. Second, we contribute to the more general literature that studies government-guaranteed loan programmes and their implications for the real economy. The existing literature points overall to the usefulness of such programmes in reducing informational costs and in dampening the effects of adverse aggregate shocks.

Section 2 describes the related literature, while Section 3 describes the COVID-19 loan programme, makes an international comparison and provides an overview of firms in Switzerland. Section 4 presents the data that are used in the empirical analysis in Section 5. Finally, Section 6 provides the conclusion.

## Literature review

Research on the Swiss COVID-19 loan programme is at this stage only nascent. We are aware of three contributions. Similar to our paper, two studies (Brülhart et al., 2020; Zoller-Rydzek and Keller, 2020) investigate the determinants of participation in government support programmes during the pandemic. In addition to the loan programme, both papers also consider other support programmes, such as short-time work. In contrast to our analysis, which is based on a comprehensive dataset of all eligible firms, these two contributions are based on surveys. More specifically, Brülhart et al. (2020) use a survey of 1011 self-employed workers and SMEs conducted in mid-April 2020.^5^ The participants in that survey were asked about the importance and their participation in three government programmes, namely (1) short-time work, (2) income replacement for self-employed workers and small business owners and (3) COVID-19 loans. Programme participation is then related to different variables measuring the extent of the lockdown as well as firm-specific economic (e.g. employment), financial (e.g. debt and profit ratio) and other (e.g. linguistic region, age and education of respondent) variables. Brülhart et al. (2020) find that lockdown restrictions are positively related with the usage of both short-time work and COVID-19 loans. However, they find that lockdown restrictions are less important for explaining the participation in the loan programme than for explaining the participation in other government support programmes. Moreover, they find that previously indebted firms are more likely to take up COVID-19 loans.

Another study based on survey evidence is the one by Zoller-Rydzek and Keller (2020), who build a theoretical model and test the resulting empirical implications by using data from the ZHAW managers barometer survey.^6^ In line with their theoretical model, they find that the pre-pandemic business situation seems to be an important driver of programme participation. In particular, firms in a worse ex ante situation are less likely to take out a COVID-19 loan. Zoller-Rydzek and Keller (2020) conclude that there seems to be no evidence that the programme creates zombie firms. In their model, a zombie firm is a firm that survives the crisis thanks to the programme but cannot repay the debt.

The third contribution by Kaufmann (2020) does not study the determinants of programme participation but investigates its effect on the macroeconomy. In particular, he analyses the impact of the COVID-19 loan programme on unemployment. He finds that higher loan supply due to the programme indeed reduces unemployment, with approximately CHF 400,000 of loan volume needed to save one job.

Apart from the aforementioned more specific literature on the Swiss COVID-19 loan programme, our paper relates to different strands of the literature relevant for government credit guarantee programmes.^7^ The unifying questions in this regard are why such a programme might be needed, which firms should be targeted and whether these programmes have been effective.

First, why might a government-guarantee loan programme be needed? There is a broad literature on financial frictions, where informational asymmetries or moral hazard and thus agency problems potentially lead to a more difficult access to credit.^8^ For instance, in the financial accelerator literature in the spirit of Bernanke and Gertler (1989) and Bernanke et al. (1999), agency costs lead to a premium on external finance and deadweight losses, while models along the line of Stiglitz and Weiss (1981) feature equilibrium rationing. Crisis situations such as the COVID-19 pandemic could lead to a sudden increase in uncertainty and informational problems, in turn increasing the difficulty to access credit or even leading to rationing. In such a situation, there might be a welfare-improving role for state guarantees as an insurance mechanism.^9^ By overcoming informational problems, the state as an entity with “deep pockets” basically acts as insurance for the entire economy.

Second, for whom might a government-guarantee loan programme be set up? The findings in the literature indicate that SMEs are particularly affected by informational issues and hence face problems in obtaining external finance. Gertler and Gilchrist (1993, 1994) study the impact of a cash squeeze on firms of different sizes and find that small firms, in contrast to larger ones, cannot use borrowing as easily to smooth cash-flow shocks. Small firms typically have less outside options of external finance. Chodorow-Reich (2014) use the more recent episode of the 2008/09 financial crisis to show that SMEs have more difficulty accessing credit during credit crunches, which in turn has negative implications for the real economy. Thus, it is not surprising that credit guarantee programmes are among the most common forms of government support for SMEs, as indicated, for instance, in Beck, Klapper, and Mendoza (2010).^10^

Third, have credit guarantee programmes worked? Overall, previous governmental loan guarantee programmes are typically found to be successful. This evaluation is carried out along several dimensions. For example, Cowling (2010) finds that small firms in the UK are indeed affected by credit rationing and that this situation can be addressed by a guarantee programme. In another study on the UK, Gonzalez-Uribe and Wang (2020) investigate the effect of a loan guarantee introduced during the 2008/09 financial crisis on firm outcomes. They find that the economic benefits significantly outweigh the costs of the programme. Riding and Haines (2001) and Riding, Madill, and Haines (2007), using the case of Canada, study the question of “additionality” of a credit guarantee programme. They ask whether such a programme leads to the extension of additional loans, which otherwise would not have been granted, or whether there is just a substitution of private loans by publicly guaranteed ones. Using credit scoring, they show that firms that otherwise would not have obtained a loan (based on the credit score) are able to secure a loan via the programme. Finally, Saito and Tsuruta (2014) analyse the costs in terms of adverse selection and moral hazard of these programmes. Their findings indicate the presence of both costs. Based on the rich public credit guarantee landscape in Japan, they show that banks with more risky customers offer more guaranteed loans. Moreover, they find that firms with guaranteed loans are more likely to default. This finding is more prevalent for guarantee programmes covering 100% than for programmes covering 80%.

## COVID-19 loan programme

On 26 March 2020, the Swiss federal government launched the COVID-19 loan guarantee programme to provide firms quick access to loans that could be used to bridge potential liquidity shortfalls resulting from the pandemic.^11^ The programme was open to the vast majority of firms residing in Switzerland; only firms with an annual turnover of more than CHF 500 million and firms founded after February 2020 could not participate.^12^

Under the programme, companies could receive from their bank government-guaranteed loans for an amount up to 10% of their annual turnover (up to a maximum of CHF 20 million) and with a maturity of five years.^13^ A first loan tranche of up to CHF 500,000 is fully guaranteed by the government. Larger companies could apply for a second tranche (called COVID-19 plus loan), of which the federal government would guarantee 85%. The pricing of the loan programme was attractive, as the first (second) tranche has an interest rate of 0% (0.5% for the guaranteed part) in the first year.^14^ Access to the loans was quick and easy since lending took place via existing client-bank relationships; the money was typically disbursed within a day. Firms did not need to have a pre-existing credit history or credit relationship—a bank account was sufficient. The period for submission of applications for the programme lasted from 26 March to 31 July 2020.

Nonetheless, there are a couple of requirements that may reduce the attractiveness of COVID-19 loans for some firms. For instance, the loan cannot be used to finance investments (other than replacement investments). Participating firms are not allowed to reimburse capital contributions or pay dividends. Moreover, COVID-19 loans cannot be used to refinance private or shareholder loans or repay intra-group loans. Likewise, there are restrictions on internal (potentially international) transactions.^15^ Firm participation was hence not obvious ex ante, particularly for larger and more complex firms.

Normally, credit creation reflects both loan supply and demand. However, we exploit the fact that due to the structure of the programme as well as the coordinated and complementary policy measures taken, participation exclusively reflects firms’ demand for emergency loans. Loan supply—in terms of programme participation—was almost perfectly elastic.^16^ Indeed, banks had basically no incentive to reject loan applications: (i) credit risk was small or even non-existent due to the government guarantee;^17^ (ii) liquidity risk was also absent due to the SNB’s COVID-19 refinancing facility (CRF), by which banks can refinance the guaranteed part of the loan at the SNB policy rate by posting the guaranteed part as collateral;^18^ (iii) regulatory constraints on banks’ balance sheets were also relaxed via the Swiss financial market supervisory authority’s (FINMA) temporary adjustment of the leverage ratio calculation and at the request of the SNB, the deactivation of the countercyclical capital buffer by the federal government.^19^

The Swiss programme has not been the only loan guarantee programme established in the face of the pandemic. Tables 13–15 in the Appendix give an overview of loan guarantee programmes set up internationally at the same time as the Swiss programme. Most programmes focus on SMEs as the most relevant beneficiaries. Similar to the maturity of the loans in Switzerland, a maturity of 5 years is quite typical. The Swiss programme closes, however, at an unusually early date. Most programmes were initially intended to be open until at least the end of 2020. The Swiss conditions in terms of the share of the loan guaranteed and interest rate are more on the generous side. An interest rate of 0% without a guarantee fee for the first tranche is at the lower end of the range and the guarantee of 100% is of course at the upper end. However, there are a couple of other countries that also offer such a comprehensive guarantee. Given these attractive terms, it is probably not surprising that the usage of the Swiss programme is considerable relative to GDP in international comparison. The Swiss programme is similar in magnitude to the US programme and to the two programmes in the UK combined. Only the programmes in Hong Kong and Italy are larger in relation to GDP.

**Fig. 1:**
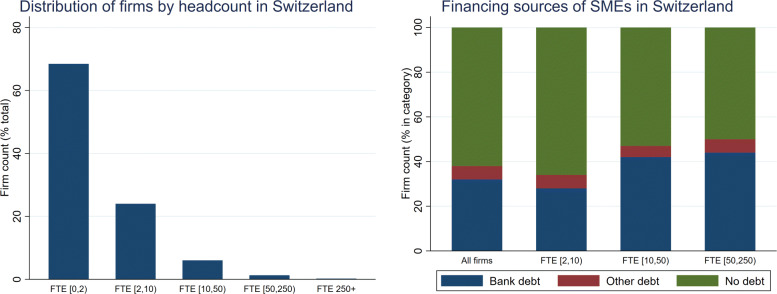
Firms in Switzerland and financing sources. *Sources*: FSO (BUR) and SECO. The graph on the left uses our set of firms, discussed in Section 4. The graph on the right uses data from a study commissioned by SECO and conducted by the Lucerne University of Applied Sciences and Arts in the fall of 2016. The survey covers 1922 SMEs in Switzerland (with less than 250 employees) and assesses their financing forms, sources and conditions. “Bank debt” typically consists of mortgages and credit lines, whereas “Other debt” contains, for example, bonds and trade credit

The COVID-19 loan programme focuses on SMEs and aims to provide quick access to bank financing. Both of those aspects are motivated by the structure of firms in Switzerland and their financing sources. Figure 1 presents the distribution of firms’ size in terms of the number of full-time equivalent employees (graph on the left) and their financing (graph on the right). The distribution of firms’ size illustrates the importance of small firms for the Swiss economy. More than 92% of firms have less than 10 employees, and over 99% have less than 250 employees, thereby fitting the definition of an SME used by the Swiss State Secretariat for Economic Affairs (SECO).^20^ Given the importance of smaller firms, it is not surprising that the programme focuses on SMEs. Moreover, an examination of the typical financing structure of firms in Switzerland indicates that a majority of SMEs do not have debt: 62% of all SMEs in Switzerland were debt-free before the pandemic. This phenomenon is most pronounced for the smallest firms with 2–10 employees: two out of three of those firms are exclusively equity financed. This share drops with increasing firm size: 50% of SMEs with 50–250 employees have some form of debt outstanding. Across all firm sizes, the dominating type of outside financing is bank debt.

Overall, the data indicate that a significant share of Swiss SMEs do not have an established credit relationship. This might be a problem if firms suddenly have to bridge liquidity shortfalls by outside finance (e.g. bank debt) and could be particularly problematic for young firms that have existed for only a couple of years. A government loan guarantee programme is a potential solution to this problem, as it eliminates credit risk and solves potential informational problems between borrowers and lenders, which otherwise might impede the extension of credit.

## Data

### Construction of dependent variable

Our analysis is based on data comprising all firms in Switzerland. We bring together two datasets: on the one hand, data from the entire registry of commerce are used (Betriebs- und Unternehmensregister, short BUR); on the other hand, data from the registry of all the COVID-19 loans, recorded by the guaranteeing organisations, are used (called the JANUS database). The entries in the two datasets are matched through a unique firm identifier, which is available in both registries. We work with an anonymised version of the matched dataset, but we do know which firms have a COVID-19 loan and which firms do not. Both datasets are cross-sectional and correspond to a snapshot at the end of the COVID-19 loan programme.^21^ Table 1 provides descriptive statistics.

**Table 1:** Descriptive statistics

	Groups	Firms	Mean	Std. dev.	Min	Max
Participation (yes/no)	–	675,111	0.15	0.36	0.00	1.00
Lockdown index (sectors within cantons)	469	674,423	0.30	0.17	0.00	0.97
Home office index (sectors within cantons)	469	674,423	0.51	0.27	0.00	1.00
Short-time work (sectors within cantons)	1118	671,713	0.20	0.15	0.00	3.50
Retail payments (sectors within cantons)	540	344,859	− 0.42	0.67	− 1.00	4.22
Virus cases (in canton)	26	675,111	0.40	0.28	0.11	1.07
Fatality cases (in canton)	26	675,111	21.01	22.08	0.00	88.90
Cash ratio, mean (headcount groups within sectors)	45	234,067	0.28	0.08	0.11	0.41
Liquidity ratio, mean (sectors)	63	530,351	1.29	0.35	0.19	2.57
Liquidity ratio, mean (sectors within cantons)	560	471,257	2.70	1.51	0.72	51.08
Liquidity ratio, median (sectors within cantons)	560	471,257	1.66	0.54	0.51	5.43
External financing (headcount groups within sectors)	18	214,489	0.39	0.11	0.28	0.67
Debt ratio, mean (sectors)	44	483,976	0.66	0.14	0.29	1.04
Debt ratio, mean (headcount groups within sectors)	54	230,420	0.41	0.17	0.21	0.87
Debt ratio, mean (sectors within cantons)	561	471,728	0.72	0.26	0.35	6.73
Debt ratio, median (sectors within cantons)	561	471,728	0.68	0.10	0.31	0.97
Profit margin, mean (headcount groups within sectors)	40	218,682	0.09	0.06	0.02	0.32
Profit to int. ratio, mean (headcount groups within sectors)	31	160,133	0.36	0.31	0.07	1.71

*Sources*: FSO (BUR), JANUS, Faber et al. (2020), SECO, SNB, FOPH, FSO, CompNet. See main text for details. The table shows the number of groups available for the variable, and the corresponding number of firms to which the group variable can be matched. The mean, standard deviation, minimum and maximum are computed for the matched firms. See main text for the variable definitions

Our cleaned dataset contains 675,111 active firms in Switzerland that were eligible for a COVID-19 loan. This set of firms is obtained by selecting entities from an initial 1.87 million entries available in the BUR registry. We excluded entries that are not active, currently in liquidation, entities without economic activity (such as investment and legal purpose vehicles) as well as domestic and foreign government entities (such as public administrations). We kept data on financial companies as well as companies operating in the primary sector (i.e. agriculture) as those companies were also eligible for a COVID-19 loan. The exclusion criteria are further detailed in Appendix “Data construction”. The BUR data also provide other information, such as the economic sector,^22^ firm size (in terms of full-time equivalent employees), firm age (via the entry date in the registry), or the canton (there are 26 cantons in Switzerland) in which the firm is legally registered.^23^ Our firm count is close to the available count of 656,364 active firms reported by the Federal Statistical Office (FSO) in January 2020. Similarly, our set of firms replicates well the economic sector, region, headcount and legal form distributions that are made available by the FSO (see Appendix “Data construction”).

**Fig. 2:**
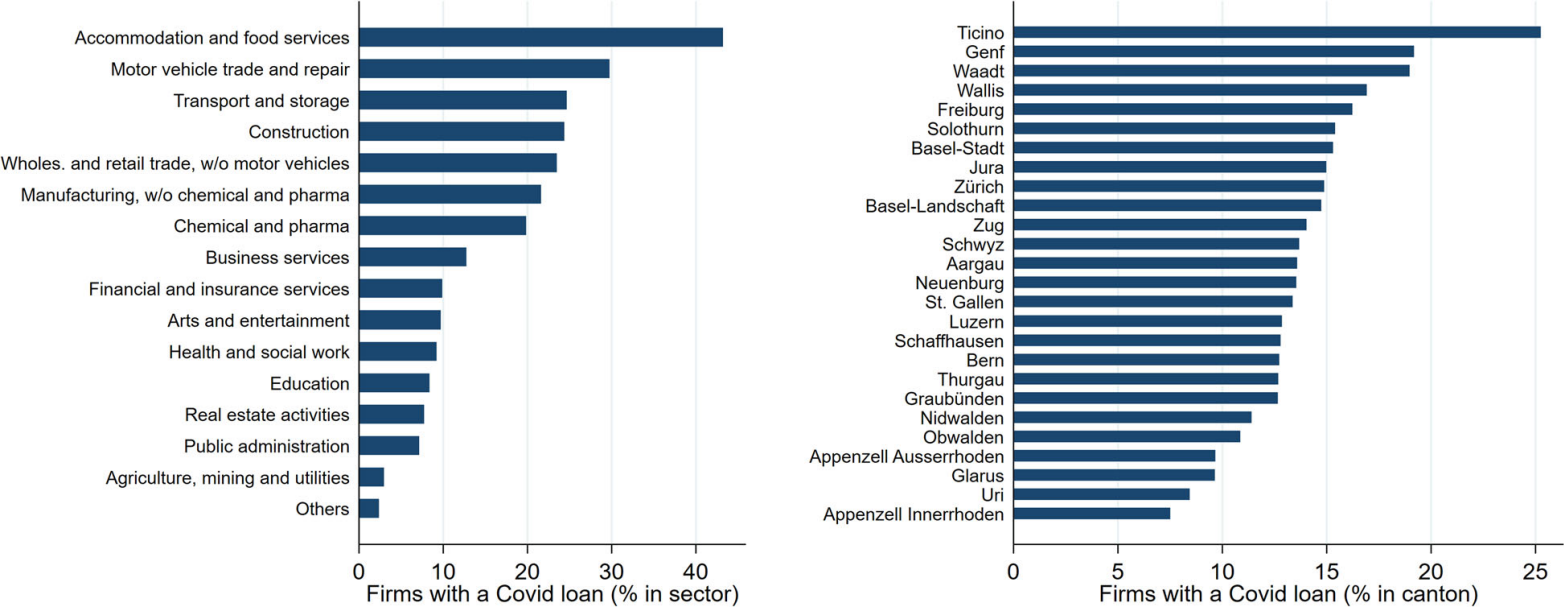
Firm participation, by economic sector and canton. *Sources*: FSO (BUR), JANUS and own calculations. The economic sectors are obtained by aggregating the more granular NOGA two-digit codes. The sector *Others* contains the activities of households as employers, the production activities of households for their own use and the activities of extra-territorial organisations and bodies

According to the latest estimate at the time of writing, there were 135,261 standard COVID-19 loans outstanding when the programme ended. This corresponds to about 20% of our firm count. We are able to match 103,605 loans to the BUR, as some loans were already paid back in full and not all loans have a unique firm identifier (see Appendix “Data construction”). Hence, we obtain by construction a lower participation rate of approximately 15%. Our data set shows that firms participated in the loan programme across sectors and cantons. Figure 2 shows the participation rates by broad economic sectors and cantons. Overall, participation across cantons is characterised by a considerable heterogeneity. By sector, the dispersion is even larger. The sector with the highest participation rate is *accommodation and food services*, with 43%. The lowest sectoral participation rates, below 3%, are found in *agriculture, mining and utilities* and in *others* (consisting of household-related production and extra-territorial organisations). Across cantons, the participation rate ranges between 7% (Appenzell Innerrhoden) and 25% (Ticino).

### Explanatory variables

The figures described above reflect how firms that operate in different sectors and regions were affected by the crisis. However, an unanswered question is what drove participation in the loan programme? We bring answers by considering three broad dimensions of loan demand, namely, a firm’s sensitivity to the lockdown, its exposure to the virus intensity, and the firm’s initial financial conditions. The different measures that we use are summarised in Table 1. Because these measures are not available at the firm level, we use group variables from various data sources; each firm is then matched to its corresponding group.

#### Sensitivity to the lockdown

Loan demand may reflect the abrupt fall in revenue implied by lockdown restrictions. Assessing a firm’s sensitivity to the lockdown is not straightforward: hence, we resort to four different measures. To ensure exogeneity, our preferred measures are a lockdown index, which relies on physical proximity, and a home office index, which relies on the possibility to perform tasks at home. Faber, Ghisletta, and Schmidheiny (2020) built the index by using the Occupational Information Network (*ONET*) survey, which asks workers questions about the level of physical proximity that is required in their occupation. Individual survey answers are translated into an index that is available for economic sectors within cantons, yielding a total of 469 groups. The index ranges between zero and one. A value of zero corresponds to little physical proximity needed, whereas a value of one indicates that physical proximity is essential to the worker’s tasks. The lowest index values are found in sectors, such as financial and insurance activities, or agriculture, whereas the highest values are found in sectors such as accommodation or construction.^24^Faber et al. (2020) also compute a home office index with the *ONET* survey. The home office index can be used as an alternative measure of lockdown restrictions.^25^ In contrast to the lockdown index, the home office index captures the possibility for a worker to perform tasks at home. A value of zero indicates that tasks cannot be operated remotely (for instance, a machine is needed), whereas a value of one implies that the worker can readily perform tasks from home. The two indices are exogenous in the sense that a firm cannot easily (or rapidly) alter the work conditions that require physical proximity for production or that allow workers producing from their homes. We complement the indices by using two indicators of business activity. First, the proportion of firms that use the Swiss short-time work scheme (or *Kurzarbeit*) in a given sector within a canton is obtained from SECO numbers relative to the firm counts in our cleaned dataset.^26^ This gives a total of 1,118 groups. Second, we obtained data on retail card payments in Switzerland from Kraenzlin, Meyer, and Nellen (2020). Based on this data, we compute the year-on-year percentage change in transaction values for April 2020. Comprising 540 groups, the data are available for sectors within cantons.

#### Exposure to virus intensity

Loan demand may also reflect the severity of the pandemic situation per se. The cautious behaviour of households (i.e. going less to shops or buying more online) may increase with the severity of the pandemic. In particular, the degree of behavioural adjustment is likely to be regional. To measure the intensity of the virus spread, we use the cumulative cases (as a percentage of the cantonal population) in the canton in which the firm is legally registered. Additionally, we use the cumulative number of fatalities due to the virus (expressed per 100,000 inhabitants). Both measures are as of 13 July for the 26 Swiss cantons and are obtained from the Federal Office of Public Health (FOPH).

#### Initial financial conditions

Loan demand may depend on a firm’s initial financial conditions. We measure liquidity and debt conditions via several group-level variables. The broader group-level liquidity variable that we have is a cash to assets ratio obtained from the CompNet survey of Swiss firms.^27^ The average ratio in 2017 is available across five headcount groups within nine sectors, representing a total of 45 groups. Additionally, we use a more granular liquidity ratio made available for the year 2018 by the FSO. In particular, the liquid asset to short-term debt ratio provided by the FSO is not only available for 63 sectors but also for sectors within cantons (totalling 560 groups). The corresponding mean and median ratios were computed by the FSO for groups that contained a minimum of five surveyed firms.^28^ External financing is measured in two ways. First, we use the proportion of firms with external financing (both bank and non-bank debt) in 2016. The data was made available for broad headcount groups within sectors and totals 18 groups.^29^ Second, we measure indebtedness via the debt to asset ratio. We use the average ratio in 2017 from CompNet; this ratio is available for 44 of the 45 headcount-sector groups mentioned above. Additionally, we use the more granular debt ratios made available for the year 2018 by the FSO. The FSO debt ratio is available for 54 sectors but also for sectors within cantons (totalling 561 groups). Again, the corresponding mean and median ratios were computed by the FSO for groups that contained a minimum of five surveyed firms.^30^ Last, we use two measures of firm profitability for further analysis in Section 5.3: the profit margin as well as the profit to interest payment ratio. The 2017 average ratios across headcount groups within sectors, are available from CompNet.

**Fig. 3:**
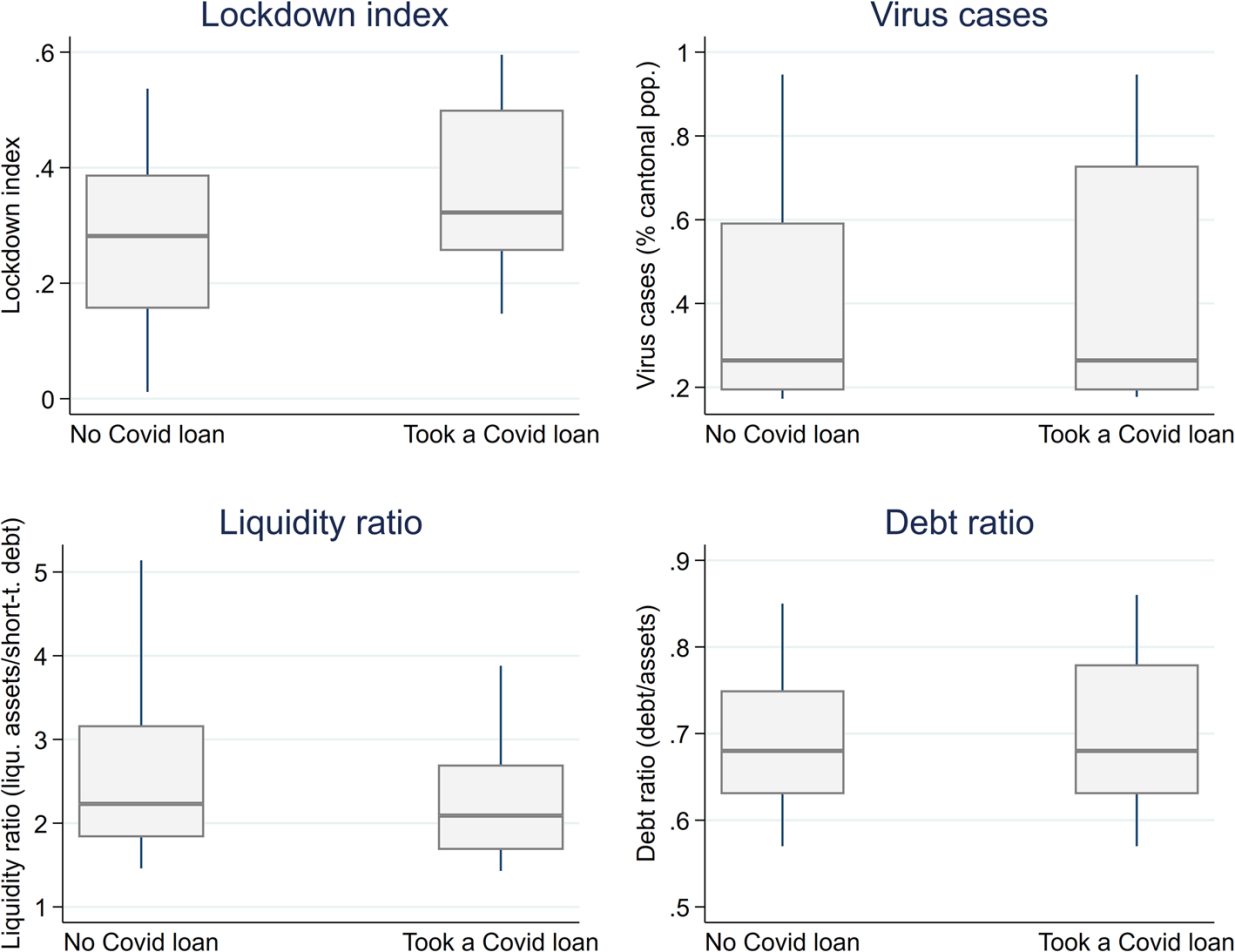
Main explanatory variables by firm participation. *Sources*: FSO (BUR), JANUS, Faber et al. (2020), SECO, SNB, FOPH, FSO, CompNet. See main text for details. *Notes*: The distribution of the variables’ values by firm participation is shown. The liquidity and debt ratios are at the sector-canton level. The median is boxed around the 25th and 75th percentiles. The whiskers are the 10th and 90th percentiles, respectively

Figure 3 shows the distribution of the main explanatory variables by firm participation in the loan programme. Firms with a COVID-19 loan tend to operate in sectors (within cantons) that are more sensitive to the lockdown; both the median and the inter-quartile range of the lockdown index for firms participating in the loan programme are higher than those for firms that do not participate in the loan programme. Likewise, firms that participate in the programme tend to be located in cantons with more virus cases. The liquidity ratio of participating firms is lower than that of non-participating firms. This holds true not only for the median liquidity ratio, but also for the inter-quartile range, which is narrower. By contrast, firm indebtedness seems to be similar across firm participation. While the debt ratio’s 75th percentile is higher for firms with a COVID-19 loan, the median does not differ from that of firms without a loan.

## Empirical analysis

To disentangle the different determinants of loan demand, we consider a standard logit model:^31^

$$\begin{array}{*{20}l} y_{i} = \frac{ \exp (\mathbf{x}_{i} ' \beta)}{ 1+ \exp (\mathbf{x}_{i} ' \beta)} + \varepsilon_{i} \quad, \end{array} $$

in which *y*_*i*_ is a binary variable that indicates the participation of firm *i* in the loan programme and **x**_*i*_ is a vector of explanatory variables. It contains the measures described above as well as dummies that control for firm size (via full-time equivalent headcount group buckets) and firm age (via firm age group buckets). By doing so, we (partly) control for firms’ individual characteristics. Our model permits the assessment of each determinant of loan demand, while keeping constant the other factors. As indicated in the regression output tables, standard errors are clustered at the level of the demand determinant (which is a group variable) that enters the regression. The coefficients of a logit regression represent the log of the odds ratio, which is hard to interpret quantitatively. Our discussion of the regression results hence focuses on the sign and the significance of the coefficients, which determine the direction of the effect. We assess the magnitude of the corresponding economic effects by plotting predictive margins.

Next, we present our regression results. The main regression results are described in Section 5.1, which discusses the firms’ sensitivity to the lockdown, their exposure to the virus intensity as well as their ex ante liquidity and indebtedness positions. Based on the various measures detailed in Section 4, more detailed results are then provided for the lockdown and virus intensity variables (Section 5.2) as well as for the financial variables (Section 5.3). Additionally, we discuss whether participation in the programme is higher for zombie firms. Next, we use the heterogeneity in our dataset in greater detail to assess whether the loan programme also reached the potentially more vulnerable firms, i.e. small and young firms (Section 5.4). Finally, additional robustness checks are reported in Section 5.5.

### Main results

Our main regression results are reported in Table 2. Columns (1)–(4) provide estimates focusing on one main variable; these estimates are cross-checked in a multivariate specification in column (5). First, we show that participation in the loan programme is affected significantly by a firm’s sensitivity to the lockdown (column 1). Firms in sectors within cantons associated with a more stringent lockdown index value have a higher probability of participation. This finding is also true for firms in cantons with more virus cases (column 2), which indicates that changes in customer behaviour were not just determined by lockdown restrictions but also by the fear of the virus. Additionally, we provide empirical support that the loan programme reached firms associated with less liquid groups. Indeed, our estimates show that firms in groups with a lower ex ante liquidity ratio have a higher probability to participate in the programme (column 3). Finally, there is no clear evidence that firms belonging to more indebted groups have a higher participation rate (column 4). In the next subsections, we use different measures to assess the robustness of these findings.

**Table 2:** Main results of the binary response model

	(1) Part.(y/n)	(2) Part.(y/n)	(3) Part.(y/n)	(4) Part.(y/n)	(5) Part.(y/n)
Lockdown index (sectors within cantons)	2.16***				1.75***
Virus cases (in canton)		0.74***			0.73***
Liquidity ratio, mean (sectors within cantons)			− 0.13***		− 0.11***
Debt ratio, mean (sectors within cantons)				− 0.06	− 0.21
Headcount dummies	Yes	Yes	Yes	Yes	Yes
Age dummies	Yes	Yes	Yes	Yes	Yes
Constant	Yes	Yes	Yes	Yes	Yes
Observations	674423	675111	471257	471728	471211
Log-likelihood	− 277189.69	− 281379.54	− 215653.56	− 216776.51	− 212285.10

Logit model. The dependent variable is a firm-level binary variable that indicates firm participation in the loan programme. Standard errors are clustered at the level of the grouped variable of interest; in column (5), clustering is at the sector-canton level of the FSO financial variables. The number of observations varies depending on data availability of the grouped variables. ***, ** and * denote statistical significance (two-tailed) at the 1%, 5% and 10% significance levels, respectively. The coefficients of the headcount and age dummies are displayed in Appendix 6

### Lockdown and virus intensity

The loan programme aimed to provide liquidity to firms whose turnover was affected by the coronavirus crisis. To evaluate whether the programme reached that objective, we assess how participation depends on firms’ exposure to the lockdown restrictions as well as to the regional virus intensity.

We find strong evidence that participation depends on a firm’s exposure to the government-imposed lockdown restrictions. Table 3 reports regression results for the sensitivity of firms to both lockdown restrictions and regional virus intensity. Columns (1) to (4) show specifications focusing on the variables measuring lockdown restrictions that were described in Section 4. Our preferred lockdown restriction variables, namely, the lockdown and the home office indices, are both statistically significant and have the expected signs. Both the physical proximity required for production and the possibility to produce from home are inherent to the type of business in which a firm operates. In that sense, the two corresponding indices are exogenous; firms cannot easily—or rapidly—alter their sensitivity to the lockdown restrictions. Column (1) shows that firm participation increases with the lockdown index: a firm (in a sector-canton group) whose production requires relatively more physical proximity is more likely to participate in the loan programme. Likewise, column (2) shows that participation decreases with the home office index. In other words, participation is higher for firms whose workers cannot execute tasks remotely.

**Table 3:** Results: Lockdown and virus intensity variables

	(1) Part.(y/n)	(2) Part.(y/n)	(3) Part.(y/n)	(4) Part.(y/n)	(5) Part.(y/n)	(6) Part.(y/n)
Lockdown index (sectors within cantons)	1.75***					
Home office index (sectors within cantons)		− 1.54***				
Short-time work (sectors within cantons)			3.00***			
Retail payments (sectors within cantons)				− 0.08		
Virus cases (in canton)					0.73***	
Fatality cases (in canton)						0.01***
Headcount dummies	Yes	Yes	Yes	Yes	Yes	Yes
Age dummies	Yes	Yes	Yes	Yes	Yes	Yes
Other demand determinants	Yes	Yes	Yes	Yes	Yes	Yes
Constant	Yes	Yes	Yes	Yes	Yes	Yes
Observations	471211	471211	470274	247969	471211	471211
Log-likelihood	− 212285.10	− 210736.48	− 206940.47	− 119304.33	− 212285.10	− 212051.60

Logit model. The dependent variable is a firm-level binary variable that indicates firm participation in the loan programme. The other demand determinants comprise the Table 2 variables (lockdown index, virus cases, liquidity ratio, debt ratio) excluding the demand determinant shown in the respective columns. Standard errors are clustered at the level of the grouped variable of interest. The number of observations varies depending on data availability of the grouped variables. ***, ** and * denote statistical significance (two-tailed) at the 1%, 5%,and 10% significance levels, respectively

**Fig. 4:**
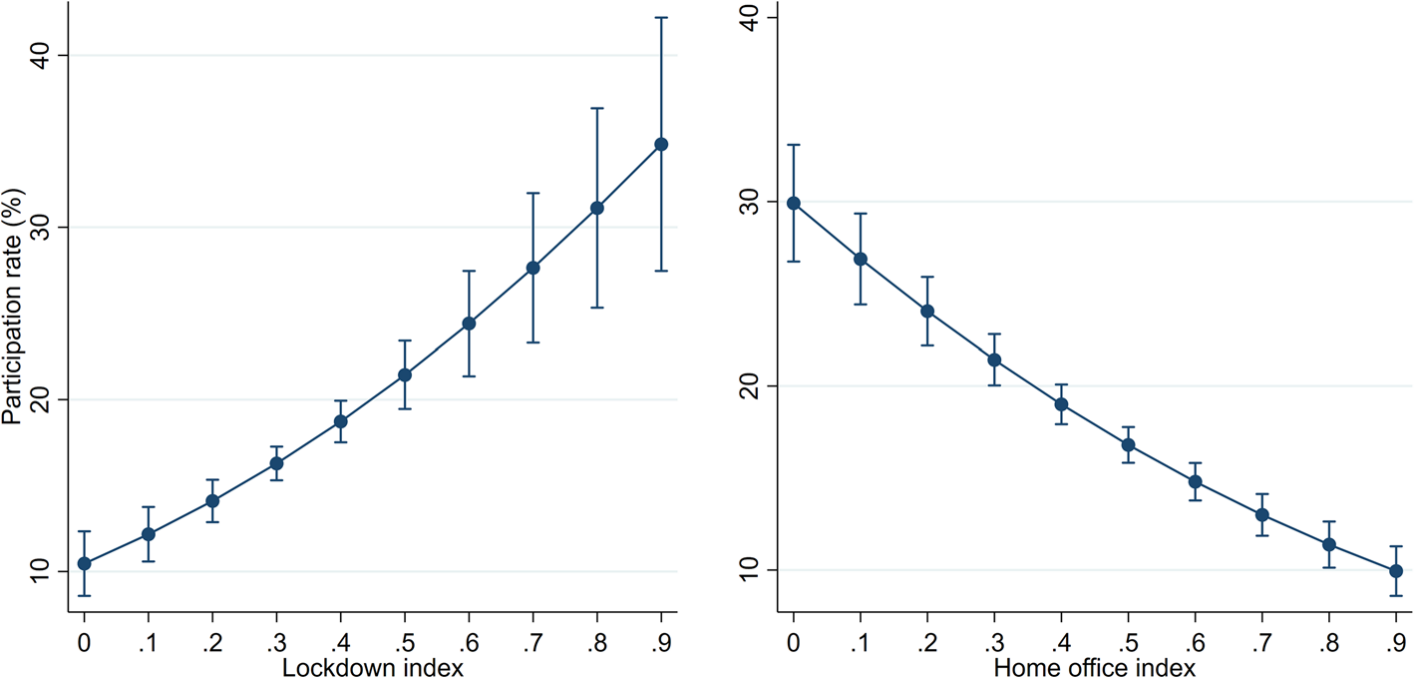
Margins of lockdown measures. Predictive margins resulting from column (5) in Table 2 are shown. The margins for the home office index use the same estimation but replace the lockdown index by the home office index. Whiskers indicate 95% confidence intervals

Importantly, the sensitivity of a firm to lockdown restrictions has a sizeable effect on participation. Figure 4 shows the predictive margins of the lockdown and home office indices. The values observed for the two indices can explain a wide interval of participation rates across sector-canton groups; this interval ranges between 10 and 35%. Participation in the loan programme is hence much higher for firms whose production was severely restricted by the lockdown.

These results are in line with the informative, although more endogenous (i.e. simultaneous), variable of short-time work (see column 3 in Table 3). Short-time work is a complement to credit guarantees, as both policy measures alleviate firm’s financial strain that results from lockdown restrictions. Intuitively, firm participation significantly increases with the use of short-time work. We find moreover that the year-on-year change in retail card payments in a sector-canton group is not significantly related to firm participation (see column 4). Solely measuring the change in retail card payments—which does neither cover all economic sectors, as indicated by the smaller sample used in column (4) nor all payment methods used—does not explain firm participation in the loan programme.

**Fig. 5:**
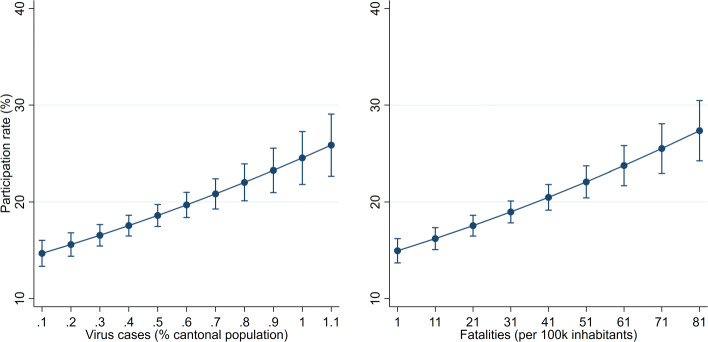
Margins of virus intensity measures. *Notes*: Predictive margins resulting from column (5) in Table 2 are shown. The margins for fatalities use the same estimation but replace virus cases by fatalities. Whiskers indicate 95% confidence intervals

A higher regional intensity of the pandemic may prompt households to become more cautious, in turn affecting nearby firms negatively. For instance, people may visit restaurants and shops less frequently to reduce the probability of getting infected. Indeed, the regional virus intensity also drives firm participation in the loan programme. In Table 3, columns (5) and (6) show that participation significantly increases in both virus cases and fatalities.^32^ A firm that operates in a canton that has a relatively high virus intensity is hence significantly more likely to take a COVID-19 loan.^33^ Despite being smaller in magnitude than the lockdown effects discussed above, the virus effect remains economically meaningful. Figure 5 plots the predictive margins for both the virus cases and fatalities. Cantonal variations in virus intensity are associated with firm participation rates that vary between 15 and 25%.

### Financial position

Now, we discuss how the ex ante liquidity and indebtedness positions affect firm participation. Additionally, we evaluate whether the loan programmes particularly attracted firms that based on their ex ante financial situation were identified as zombies.

The goal of the programme was to provide firms with adequate liquidity. One measure of success is accordingly whether the programme reached firms in groups with relatively weaker ex ante liquidity positions. Firms with less liquidity before the pandemic are more likely to end up in a precarious position once the effects of the pandemic have played out. We assess this by estimating how ex ante liquidity affects participation. Table 4 provides regression results using the various liquidity measures detailed in Section 4. Three out of four coefficients are statistically significant, and all coefficients have a negative sign. Hence, we find evidence that firms in groups with lower liquidity ratios have a higher probability of participating in the programme. The table orders variables by increasing granularity. Column (1) uses the cash ratio based on the CompNet data. This relatively coarse measure, which uses variations across headcount groups within sectors (45 groups), is only available for about 30% of the firms in our sample. The effect of this measure on participation is not statistically significant. In columns (2)–(4), we use the more granular measures from the FSO. Column (2) reports the results for the liquidity ratio at the sector level (63 groups). Columns (3) and (4) use the liquidity ratio at the finer sector-canton level (560 groups), and this ratio is based on the prevailing average and median ratios, respectively. For these three more granular measures, the effect of liquidity on firm participation is statistically significant.

**Table 4:** Results: Liquidity variables

	(1) Part.(y/n)	(2) Part.(y/n)	(3) Part.(y/n)	(4) Part.(y/n)
Cash ratio, mean (headcount groups within sectors)	− 0.22			
Liquidity ratio, mean (sectors)		−0.84**		
Liquidity ratio, mean (sectors within cantons)			−0.11***	
Liquidity ratio, median (sectors within cantons)				−0.15**
Headcount dummies	Yes	Yes	Yes	Yes
Age dummies	Yes	Yes	Yes	Yes
Other demand determinants	Yes	Yes	Yes	Yes
Constant	Yes	Yes	Yes	Yes
Observations	205695	467375	471211	471211
Log-likelihood	−117922.97	−208789.85	−212285.10	−212821.58

Logit model. The dependent variable is a firm-level binary variable that indicates firm participation in the loan programme. The number of observations varies depending on data availability of the grouped variables. The other demand determinants comprise the Table 2 variables (lockdown index, virus cases, liquidity ratio, debt ratio) excluding the demand determinant shown in the respective columns. Standard errors are clustered at the level of the grouped variable of interest. The number of observations varies depending on data availability of the grouped variables. ***, ** and * denote statistical significance (two-tailed) at the 1%, 5% and 10% significance levels, respectively

**Fig. 6:**
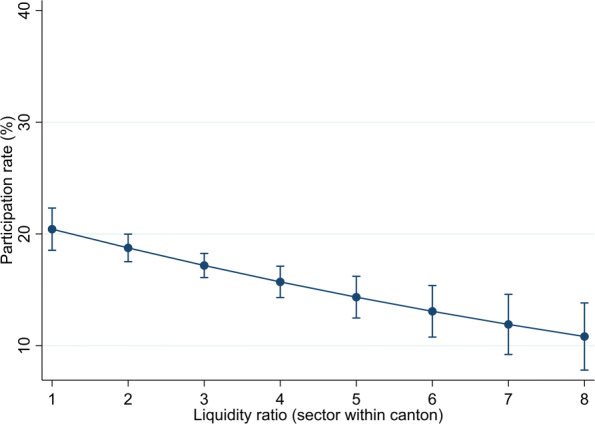
Margins of liquidity measure. Predictive margins resulting from column (5) in Table 2 are shown. Whiskers indicate 95% confidence intervals

The liquidity position can also explain to a meaningful extent firm participation. To analyse the magnitude of the effect, Fig. 6 plots the predictive margins for the liquidity ratio at the sector-canton level. The range of liquidity ratios observed in our data yields participation rates ranging between around 10% to values somewhat higher than 20%. Thus, the effect of liquidity is comparable in magnitude to that of virus intensity but smaller than the effect of lockdown measures.

Next, we discuss the effect of indebtedness on firm participation. Firms associated with groups with higher leverage may face difficulties in obtaining additional non-guaranteed loans. Indebtedness may hence be positively related to firm participation in the loan programme.

Table 5 provides regression results for the indebtedness measures detailed in Section 4. As similarly done for liquidity, the indebtedness variables are ordered in increasing granularity. Overall, we find mixed evidence that indebtedness affects participation. Three out of the five indebtedness variables have a positive coefficient, indicating that more indebted firms tend to have a higher participation rate. However, the coefficient is statistically significant for only one out of the five measures.

**Table 5:** Results: Indebtedness variables

	(1) Part.(y/n)	(2) Part.(y/n)	(3) Part.(y/n)	(4) Part.(y/n)	(5) Part.(y/n)
External financing (headcount groups within sectors)	0.98				
Debt ratio, mean (headcount groups within sectors)		1.48***			
Debt ratio, mean (sectors)			0.76		
Debt ratio, mean (sectors within cantons)				− 0.21	
Debt ratio, median (sectors within cantons)					− 0.31
Headcount dummies	Yes	Yes	Yes	Yes	Yes
Age dummies	Yes	Yes	Yes	Yes	Yes
Other demand determinants	Yes	Yes	Yes	Yes	Yes
Constant	Yes	Yes	Yes	Yes	Yes
Observations	174421	202475	428690	471211	471211
Log-likelihood	− 99987.30	− 115710.68	− 196882.14	− 212285.10	− 212260.06

Logit model. The dependent variable is a firm-level binary variable that indicates firm participation in the loan programme. The number of observations varies depending on data availability of the grouped variables. The other demand determinants comprise the Table 2 variables (lockdown index, virus cases, liquidity ratio, debt ratio) excluding the demand determinant shown in the respective columns. Standard errors are clustered at the level of the grouped variable of interest. The number of observations varies depending on data availability of the grouped variables. ***, ** and * denote statistical significance (two-tailed) at the 1%, 5% and 10% significance levels, respectively

Column (1) in Table 5 shows the results based on the proportion of firms that use external financing. The coefficient is not statistically significant for this measure, which relies on variation across 18 headcount-sector groups. By contrast, the effect turns positive and statistically significant for the average debt ratio across the finer 44 headcount-sector groups used in column (2). Column (3) shows that significance drops when using instead the average debt ratio across sectors, which increases the number of observations considerably and provides more information due to a higher number of groups. Columns (4) and (5) show the results for the more granular measures that are at the sector-canton level. When using these more refined measures, which comprise observations for 561 groups, the debt ratio is not statistically significant. This finding may imply that less granular variables just reflect broader effects instead of the underlying relationship between indebtedness and participation. Accordingly, we find mixed results for the effect of indebtedness on firm participation. Significance vanishes as the indebtedness measure becomes more granular; hence, we do not illustrate the magnitude of the effect via predictive margins.

Easy access to the programme may allow pre-existing zombie firms to obtain a COVID-19 loan, which may not be economically desirable. Firms with low profitability and high leverage bind resources that cannot be relocated towards more productive activities and hence tend to dampen economic growth (see Andrews and Petroulakis (2019) for empirical evidence in the Euro area). Section 3 discussed how easy it is to access the COVID-19 loan programme. In light of our mixed results on indebtedness and against the backdrop of the different results found in the literature (see Section 2), it is natural to investigate whether the programme enables firms with low profitability and high leverage to access additional credit. To address this, we analyse how profitability measures interacted with indebtedness levels affect participation in the loan programme.

We do not find a stronger participation from firms that are associated with groups that may qualify as pre-existing zombies. To measure profitability, we use the profit margin and the profit to interest payment ratio from CompNet. The debt ratio is also available for the same headcount-sector groups from CompNet. We set a dummy variable to one when a firm belongs to a group that is highly indebted and has a low profitability. A group is considered highly indebted when the debt ratio is higher than the median (alternatively, the 75%). Likewise, a group is assigned a low profitability when the profitability measures are lower than the median (alternatively, the 25%).^34^ Table 6 shows the regressions results. The coefficients of all of the corresponding interaction terms are not statistically significant. Hence, we find no evidence of higher participation of our—admittedly crudely identified—zombie firms.

**Table 6:** Results: Zombies

	(1) Part.(y/n)	(2) Part.(y/n)	(3) Part.(y/n)	(4) art.(y/n)
Dummy: debt > median, profit margin < median	0.09			
Dummy: debt > p(75), profit margin < p(25)		0.14		
Dummy: debt > median, profit-interest ratio < median			0.16	
Dummy: debt > p(75), profit-interest ratio < p(25)				0.36
Headcount dummies	Yes	Yes	Yes	Yes
Age dummies	Yes	Yes	Yes	Yes
Constant	Yes	Yes	Yes	Yes
Lockdown index	Yes	Yes	Yes	Yes
Virus cases	Yes	Yes	Yes	Yes
Liquidity ratio	Yes	Yes	Yes	Yes
Debt ratio	Yes	Yes	Yes	Yes
Observations	192757	192757	141993	141993
Log-likelihood	− 112469.57	− 112464.19	− 85247.03	− 85121.55

Logit model. The dependent variable is a firm-level binary variable that indicates firm participation in the loan programme. The number of observations varies depending on data availability of the grouped variables. Standard errors are clustered at the level of the grouped variable of interest. ***, ** and * denote statistical significance (two-tailed) at the 1%, 5% and 10% significance levels, respectively

### Reaching vulnerable firms

One measure of success of an emergency loan programme is whether it reached firms for which access to credit is difficult. As discussed in Section 2, younger and smaller firms are likely to be financially more vulnerable—particularly during a crisis. Due to a limited track record and credit history, agency problems (informational asymmetries and, consequently, moral hazard) are typically higher for those firms, making access to external finance more difficult, regardless of whether the financing is in the form of bank loans or other forms of financing. As documented for Switzerland in Section 3, the lack of external finance might both be a result of the aforementioned problems as well as an impediment to the access to external finance in an emergency (e.g. due to the lack of an established credit relationship).

To assess whether the COVID-19 loan programme also reached potentially more vulnerable firms, we interact our explanatory variables with firm age and firm size. We measure a firm’s age by the number of elapsed years since the firm entered the registry of commerce. Firm size is measured via the headcount in full-time equivalent (FTE) employees. The regression results for firm age and size are reported in Tables 7 and 8, respectively.

**Table 7:** Results: Firm age interactions

	(1) Part.(y/n)	(2) Part.(y/n)	(3) Part.(y/n)	(4) Part.(y/n)
Age <1× Interacted measure	2.33***	0.23*	− 0.16***	− 0.21
Age [1,5)× Interacted measure	2.50***	0.46***	− 0.15***	− 0.32*
Age [5,10)× Interacted measure	1.74***	0.75***	− 0.15***	− 0.29
Age 10+× Interacted measure	1.24***	0.92***	− 0.07**	− 0.09
Headcount dummies	Yes	Yes	Yes	Yes
Age dummies	Yes	Yes	Yes	Yes
Other demand determinants	Yes	Yes	Yes	Yes
Constant	Yes	Yes	Yes	Yes
Observations	471211	471211	471211	471211
Log-likelihood	− 212094.74	− 212153.96	− 212195.25	− 212268.08
Interacted measure	Lockdown index	Virus cases	Liquidity ratio	Debt ratio

Logit model. The dependent variable is a firm-level binary variable that indicates firm participation in the loan programme. The interacted measures are listed in the last line of the table. Age is measured in years since the firm entered the registry of commerce. The first age group (*Age* <1) is the reference group for the coefficient of the chosen interacted variable. The coefficients of the other age groups consist of this reference coefficient plus the interaction term of the given age group. The other demand determinants comprise the Table 2 variables (lockdown index, virus cases, liquidity ratio, debt ratio) excluding the chosen interacted variable shown in the respective columns. Standard errors are clustered at the level of the grouped variable of interest. The number of observations varies depending on data availability of the grouped variables. ***, ** and * denote statistical significance (two-tailed) at the 1%, 5% and 10% significance levels, respectively.

**Table 8:** Results: Firm size interactions

	(1) Part.(y/n)	(2) Part.(y/n)	(3) Part.(y/n)	(4) Part.(y/n)
FTE [0,10)× Interacted measure	1.68***	0.70***	− 0.11***	− 0.25
FTE [10,50)× Interacted measure	2.01***	1.08***	− 0.11***	0.06
FTE [50,250)× Interacted measure	2.77***	0.71**	− 0.12**	0.14
FTE 250+× Interacted measure	1.70*	0.99***	− 0.23**	0.41
Headcount dummies	Yes	Yes	Yes	Yes
Age dummies	Yes	Yes	Yes	Yes
Other demand determinants	Yes	Yes	Yes	Yes
Constant	Yes	Yes	Yes	Yes
Observations	471211	471211	471211	471211
Log-likelihood	− 212260.43	− 212245.70	− 212283.71	− 212267.28
Interacted measure	Lockdown index	Virus cases	Liquidity ratio	Debt ratio

Logit model. The dependent variable is a firm-level binary variable that indicates firm participation in the loan programme. The interacted measures are listed in the last line of the table. Firm size is measured in FTE employees. The first headcount group (*FTE*[0,10)) is the reference group for the coefficient of the chosen interacted variable. The coefficients of the other headcount groups (in FTE) consist of this reference coefficient plus the interaction term of the given headcount group. The other demand determinants comprise the Table 2 variables (lockdown index, virus cases, liquidity ratio, debt ratio) excluding the chosen interacted variable shown in the respective columns. Standard errors are clustered at the level of the grouped variable of interest. The number of observations varies depending on data availability of the grouped variables. ***, ** and * denote statistical significance (two-tailed) at the 1%, 5% and 10% significance levels, respectively

**Fig. 7:**
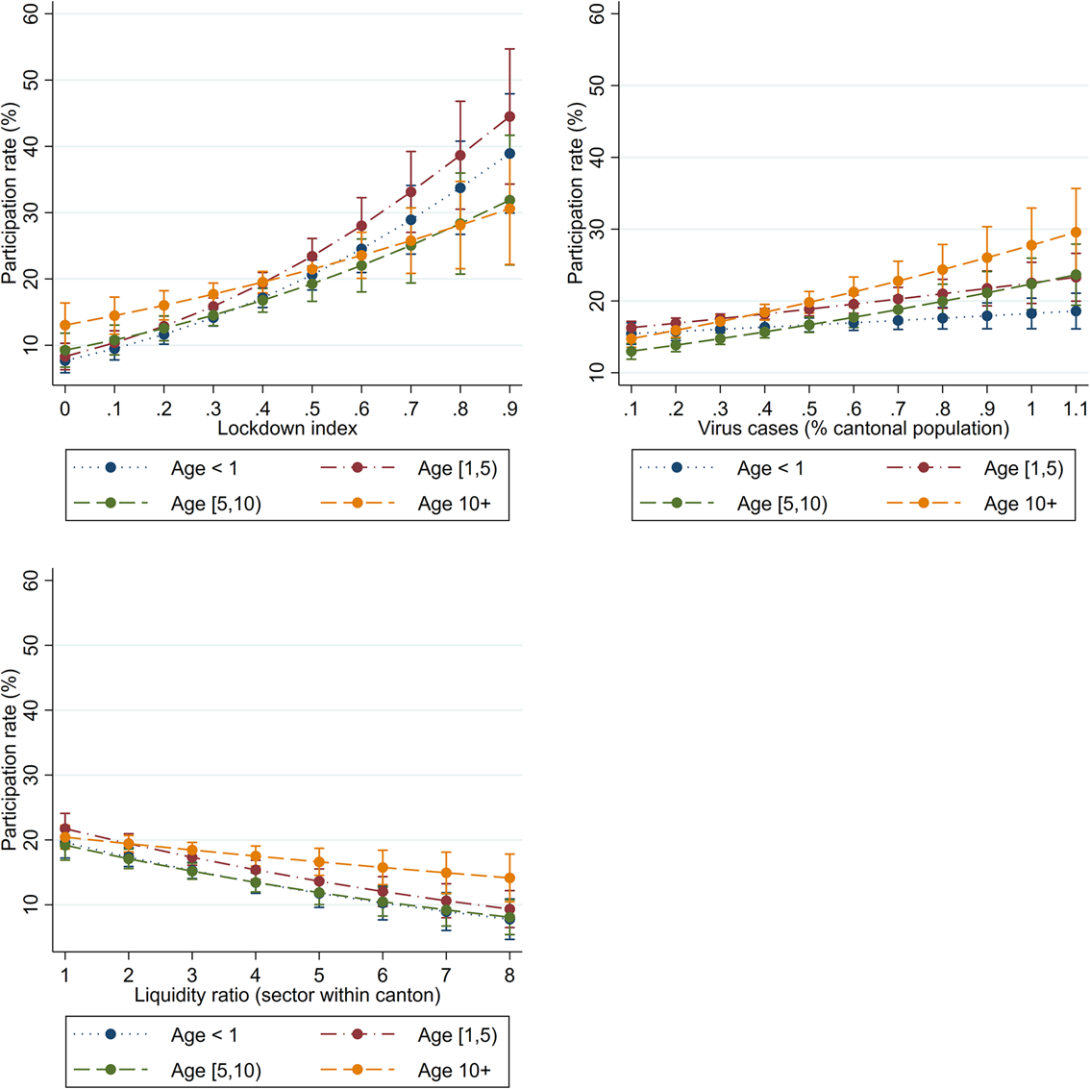
Margins of key measures, by firm age. Predictive margins resulting from Table 7 are shown. Whiskers indicate 95% confidence intervals

**Fig. 8:**
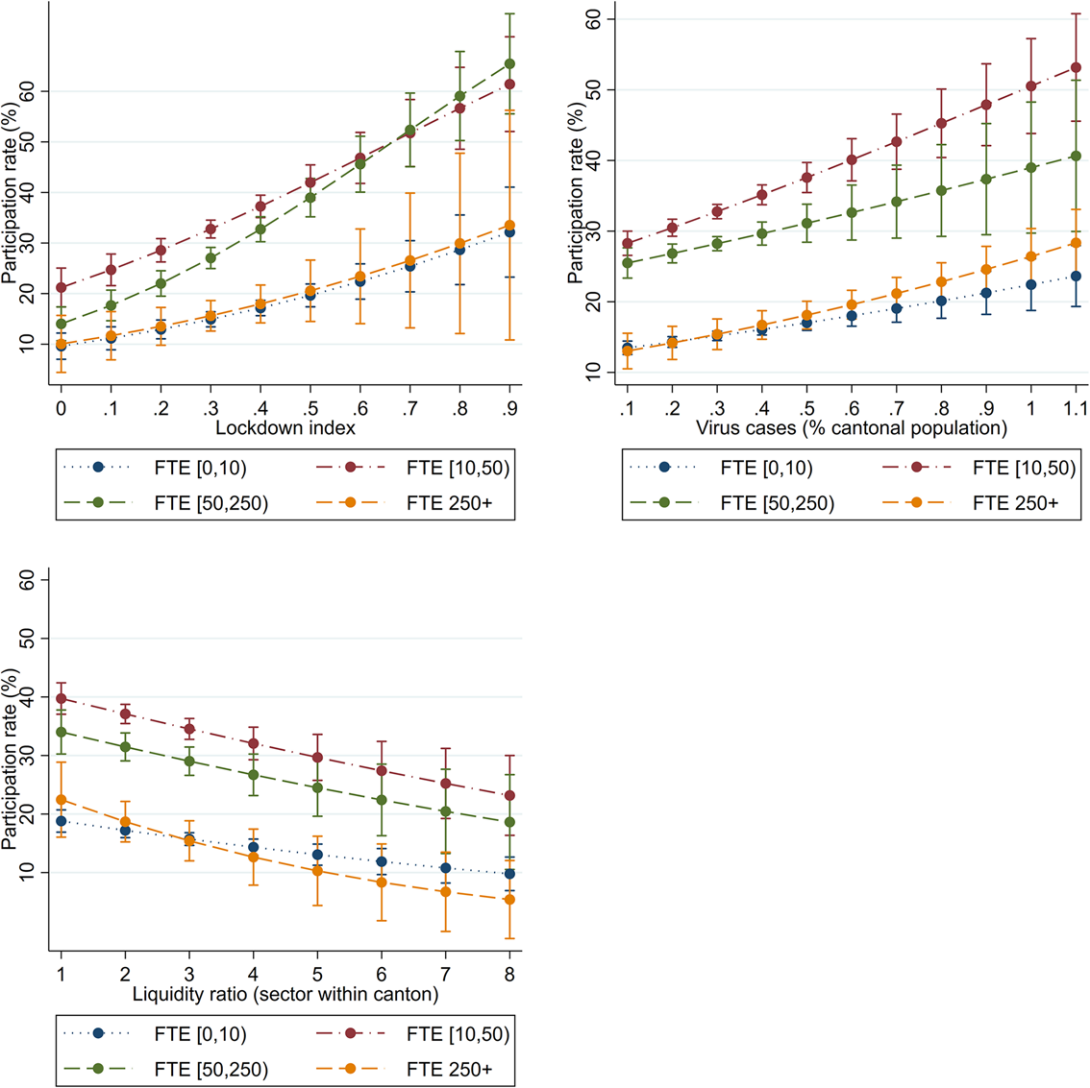
Margins of key measures, by firm size. Predictive margins resulting from Table 8 are shown. Whiskers indicate 95% confidence intervals

We find evidence that the COVID-19 loan programme reached firms that are potentially more vulnerable. Table 7 shows that the demand determinants significantly affect the participation of each age group, including the youngest firms (those that were created less than a year ago). As coefficients cannot be directly compared in a logit model, we present the conditional participation rates in Fig. 7. The 95% confidence intervals of the predictive margins overlap across age groups. For a given exposure to lockdown restrictions and virus intensity or for a given ex ante liquidity position, the probability that a firm participates in the loan programme is the same regardless of how old the firm is. Table 8 shows that the demand determinants significantly affect the participation of all size groups, including that of the smallest firms (those with less than 10 FTE employees). Figure 8 shows the predictive margins by firm size. Similarly to firm age, across firm sizes, the corresponding sensitivities do not differ. While participation levels are significantly different across some headcount groups, the sensitivity to the explanatory variables (that is, the slope of the margins) is broadly similar.

### Robustness

**Fig. 9:**
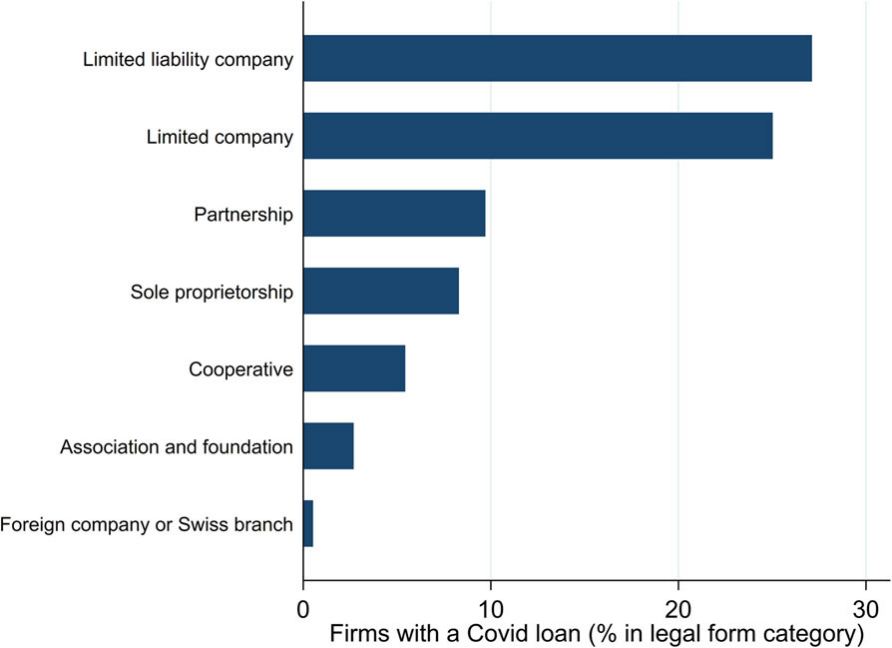
Participation rate, by legal form. *Sources*: FSO (BUR), JANUS and own calculations. The legal forms are those prevailing in Switzerland

We provide three robustness checks. First, our findings are robust in smaller subsamples. Tables 9 and 10 show the regression results for subsamples based on firm age and firm size groups. Estimating with subsamples is more restrictive than estimating with the interaction terms used in the previous subsection. With only a few exceptions, the variables of interest remain significant and the corresponding coefficient signs are unchanged for the subgroups. Second, our estimates are not affected when controlling via fixed effects for cantons and sectors. While adding interacted canton-sector dummies would by construction remove the variations exploited in our explanatory variables, we show in Table 11 that canton dummies do not affect the estimates that rely on sectoral variations (that is, the lockdown index and financial conditions). Likewise, adding dummy variables for sectors does not affect the estimate of virus intensity, which uses cantonal variations. Third, as participating firms are not allowed to pay dividends, the participation rate of limited companies might be lower than that for firms with other legal forms. However, as shown by Fig. 9, this is not the case.

**Table 9:** Robustness in subsamples, by firm age

	(1) Part.(y/n)	(2) Part.(y/n)	(3) Part.(y/n)	(4) Part.(y/n)
Lockdown index (sectors within cantons)	2.20***	2.42***	1.73***	1.29***
Virus cases (in canton)	0.22	0.49***	0.75***	0.89***
Liquidity ratio, mean (sectors within cantons)	− 0.16***	− 0.14***	− 0.14***	− 0.07**
Debt ratio, mean (sectors within cantons)	− 0.30	− 0.40**	− 0.33	− 0.04
Constant	Yes	Yes	Yes	Yes
Headcount dummies	Yes	Yes	Yes	Yes
Age dummies	Yes	Yes	Yes	Yes
Observations	12023	128230	130435	200517
Log-likelihood	− 5087.42	− 56872.18	− 53028.92	− 96746.35
Sample	Age <1	Age [1,5)	Age [5,10)	Age 10+

Logit model. The dependent variable is a firm-level binary variable that indicates firm participation in the loan programme. Standard errors are clustered at the sector-canton level of the FSO financial variables. ***, ** and * denote statistical significance (two-tailed) at the 1%, 5% and 10% significance levels, respectively

**Table 10:** Robustness in subsamples, by firm size

	(1) Part.(y/n)	(2) Part.(y/n)	(3) Part.(y/n)	(4) Part.(y/n)	(5) Part.(y/n)
Lockdown index (sectors within cantons)	1.75***	1.67***	2.03***	2.86***	1.51*
Virus cases (in canton)	0.73***	0.69***	1.08***	0.75***	0.95***
Liquidity ratio, mean (sectors within cantons)	− 0.11***	− 0.11***	− 0.10***	− 0.07*	− 0.22*
Debt ratio, mean (sectors within cantons)	− 0.21	− 0.24	0.03	0.11	0.15
Constant	Yes	Yes	Yes	Yes	Yes
Headcount dummies	Yes	Yes	Yes	Yes	Yes
Age dummies	Yes	Yes	Yes	Yes	Yes
Observations	471211	432440	30809	6828	1128
Log-likelihood	− 212285.10	− 188085.77	− 19509.86	− 3951.71	− 502.12
Sample	All firms	FTE [0,10)	FTE [10,50)	FTE [50,250)	FTE 250+

Logit model. The dependent variable is a firm-level binary variable that indicates firm participation in the loan programme. Standard errors are clustered at the sector-canton level of the FSO financial variables. ***, ** and * denote statistical significance (two-tailed) at the 1%, 5% and 10% significance levels, respectively

**Table 11:** Robustness with canton and sector dummies

	(1) Part.(y/n)	(2) Part.(y/n)	(3) Part.(y/n)
Lockdown index (sectors within cantons)	1.75***	1.82***	− 0.26
Virus cases (in canton)	0.73***	1.17	0.66***
Liquidity ratio, mean (sectors within cantons)	− 0.11***	− 0.12***	0.01
Debt ratio, mean (sectors within cantons)	− 0.21	− 0.19	− 0.10
Constant	Yes	Yes	Yes
Headcount dummies	Yes	Yes	Yes
Age dummies	Yes	Yes	Yes
Canton dummies	No	Yes	No
Sector dummies	No	No	Yes
Observations	471211	471211	471211
Log-likelihood	− 212285.10	− 211706.62	− 204289.58

Logit model. The dependent variable is a firm-level binary variable that indicates firm participation in the loan programme. Standard errors are clustered at the sector-canton level of the FSO financial variables. Sector dummies use the sectoral breakdown of the lockdown index. ***, ** and * denote statistical significance (two-tailed) at the 1%, 5% and 10% significance levels, respectively

## Conclusions

We analyse the determinants of firm participation in the Swiss COVID-19 loan programme by using a comprehensive dataset. Overall, 20% of all firms applied for a COVID-19 loan, resulting in a sizeable programme of 2.4% of GDP.

Our key findings for firm participation are as follows. First, the exposure of the firm to lockdown restrictions and the intensity of the virus in the specific region are important determinants of participation. Second, we show that firms associated with lower liquidity ratios had a significantly higher probability of participating in the programme. Third, we find no clear evidence that firm indebtedness affects participation in the programme and no evidence that pre-existing zombie firms participated more strongly in the loan programme. Fourth, we show that the programme reached all firms, including younger and smaller firms, which could be financially more vulnerable, as they are less likely to obtain outside finance during a crisis. In light of these findings, we conclude that given its stated objective, the programme seems to have been successful.

Our analysis is intended to contribute to a broader understanding of the economic measures that were taken by governments during the COVID-19 crisis. Given the potentially far-reaching implications of such large-scale policy measures, further empirical and theoretical research in this area is essential. For example, the impact of the programme on firm (e.g. profits, employment, and survival) and macroeconomic outcomes could be studied after some time has passed and more reliable data on actual outcomes become available. As another example, the role of firm networks (supply chains etc.) might be analysed with regard to participation. We leave these and further questions for future research.

## \thelikesection Appendix

### \thelikesubsection Data construction

*Registry of commerce.* Firms are selected from the 1.87 million entries in the registry of commerce (Betriebs- und Unternehmensregister, short BUR).^35^ This is done by excluding the following entries:

- Administratively and statistically non-active entries,
- Entries with a non-definitive unique identifier (UID),
- Non-relevant firm types (investment vehicles, legal purpose entities, foreign and domestic government entities, government companies),
- Non-relevant enterprise types (administrative link or VAT units, public sector, public enterprise without personnel, public sector administrative unit, enterprise owned by foreign state, foreign units without employment, errors),
- Entities currently in liquidation or in bankruptcy,
- Entities without information on the canton, economic sector (NOGA two-digit code), headcount (groups), or entry date in the registry (hence firm age).

**Fig. 10:**
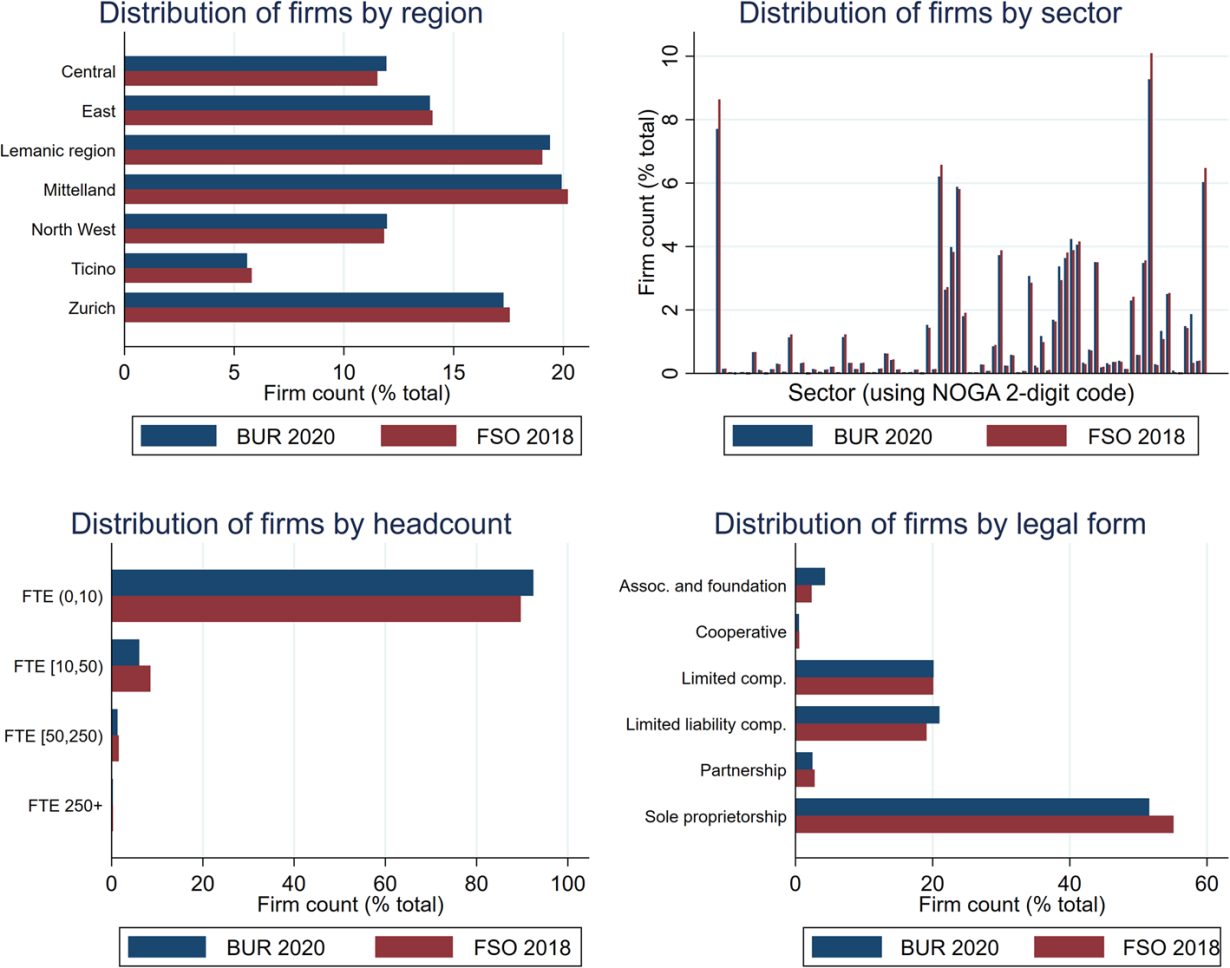
Comparison with available firm distributions. The charts compare the final sample (BUR) to the distributions made available by the FSO in 2018. Firm counts for which data are available in the categories defined by the FSO are used. Headcounts use full-time equivalent (FTE) employees

Figure 10 in Appendix shows that the resulting sample replicates well the firm distributions by region, sector, headcount group and legal form; these distributions are available for 2018 from the FSO.

*Register of COVID-19 loans.* Firms that participated in the COVID-19 loan programme are contained in the JANUS database developed by the Swiss guaranteeing institutions. Most firms can be matched to the registry of commerce via a unique identifier. Additionally, we match firms that obtained a COVID-19 plus loan, as these larger firms must take the standard COVID-19 loan as a first tranche. Out of the 135,261 firms that took a COVID-19 loan by the end of the programme (end of July 2020), 103,605 firms can be matched to the register of commerce. The discrepancy is due to two reasons. First, not all unique identifiers can be reconciled. Second, the JANUS database continues to be updated; some (3990) loans were either already paid back in full or not yet entered in the database September version that we use (which contains 131,271 loans).

**Table 12:** Main results, all coefficients displayed

	(1) Part.(y/n)	(2) Part.(y/n)	(3) Part.(y/n)	(4) Part.(y/n)	(5) Part.(y/n)
Lockdown index (sectors within cantons)	2.16***				1.75***
Virus cases (in canton)		0.74***			0.73***
Liquidity ratio, mean (sectors within cantons)			−0.13***		−0.11***
Debt ratio, mean (sectors within cantons)				−0.06	−0.21
FTE [10,50)	1.14***	1.23***	1.06***	1.07***	1.06***
FTE [50,250)	0.89***	0.88***	0.72***	0.72***	0.80***
FTE 250+	0.23*	0.20**	0.00	0.02	0.06
Age [1,5)	0.20***	0.20***	0.13***	0.13***	0.15***
Age [5,10)	0.02	−0.00	−0.05	−0.07*	−0.02
Age 10+	0.17***	0.18***	0.15***	0.14***	0.18***
Constant	− 2.63***	−2.26***	−1.38***	−1.67***	−2.27***
Observations	674423	675111	471257	471728	471211
Log-likelihood	−277189.69	−281379.54	−215653.56	−216776.51	−212285.10

Logit model. The dependent variable is a firm-level binary variable that indicates firm participation in the loan programme. Standard errors are clustered at the level of the grouped variable of interest; in column (5), clustering is at the sector-canton level of the FSO financial variables. The number of observations varies depending on data availability of the grouped variables. ***, ** and * denote statistical significance (two-tailed) at the 1%, 5% and 10% significance levels, respectively. Headcount is measured in full-time equivalent (FTE) employees, and firm age is in years. The base group has FTE [0,10) and age <1

### \thelikesubsection Main results with all coefficients displayed

**Table 13:** Loan guarantee programmes internationally

Jurisdiction	Beneficiary	Guarantee/ maximum loan size	Closing date	Interest rate	Loan maturity	Usage (CHF bn)	Usage (% of GDP)
Switzerland	Firms with turnover below CHF 500m	100% up to CHF 500’000, 85% up to CHF 20m; maximum of 10% of annual turnover	31 Jul 2020	0% interest rate up to CHF 500’000; part over CHF 500’000: 0.5% plus a bank specific rate on the remaining 15% of the loan	5 (+2) years	16.9	2.4%
Australia (Coronavirus SME Guarantee Scheme)	SME	50%/ AUD 250’000	30 Sep 2020	Initial 6-month interest holiday; rate decided by lender	Up to 3 years	N/A	N/A
Canada (Canada Emergency Business Account, CEBA)	Small businesses and non-profits	100%/ CAD 40’000	N/A	0% interest rate, no fees or principal repayments until end-2022, then 5% interest rate	Up to 5 years	20.6	1.3%
France (Bpifrance)	All firms	70–90% (higher for smaller firms); maximum of 25% of 2019 revenue or two years of payrolls	31 Dec 2020	No payment in the first year; interest rate set by the bank, guarantee cost ranging b/w 25–200 bp	Repay by end-2020, or extended by maximum of 5 year	6.5	0.3%

*Source*: Baudino (2020) and national sources

**Table 14:** Loan guarantee programmes internationally (cont.)

Jurisdiction	Beneficiary	Guarantee/ maximum loan size	Closing date	Interest rate	Loan maturity	Usage (CHF bn)	Usage (% of GDP)
Germany (Bundesregelung Kleinbeihilfen 2020)	SME	100% for loans up to: EUR 500’000 for firms with 50 employees; EUR 800’000 for others	31 Dec 2020	Individual loan rate determined by bank	N/A	5.0 (after 100 days)	0.1%
Germany (Kreditanstalt für Wiederaufbau, KfW)	All firms	90% for SME, 80% for others; EUR 1bn per company	31 Dec 2020	Subsidised loan rate (lower for SME)	Up to 5 years	58.1	1.5%
Hong Kong SAR (Special Financing Guarantee Scheme, SFGS)	SME	100%; up to total amount of employee wages and rents for six months or HKD 4m	Avaiable for 12 months	Optional principal moratorium for 1 year; rate is Prime Rate minus 2.5%; no guarantee fees	Up to 3 years	10.9	3.2%
Italy (Fondo di Garanzia PMI)	SME	80–90%: loans up to EUR 1.5m; 100%: loans up to EUR 800’000	17 Dec 2020	Guarantee cost waived; loan rates set by lenders	N/A	87.9	4.6%
Italy (Cassa Depositi e Prestiti)	All firms (SME must first apply for SME plan)	70–90%; maximum of 25% of 2019 revenues or twice payroll costs	31 Dec 2020	Individual loan rate determined by bank	6 years	N/A	N/A

*Source*: Baudino (2020) and national sources

**Table 15:** Loan guarantee programmes internationally (cont.)

Jurisdiction	Beneficiary	Guarantee/ maximum loan size	Closing date	Interest rate	Loan maturity	Usage (CHF bn)	Usage (% of GDP)
Spain (Instituto de Credito Oficial)	All firms	60–80% depending on company size and new/renewed loan); no explicit maximum	30 Sep 2020	Guarantee fees of 20–120 bp (to be borne by the bank)	Up to 5 years	N/A	N/A
United Kingdom (Coronavirus Business Interruption Loan Scheme, CBILS)	SME	100% up to GBP 250’000; 80% above GBP 250’000; up to GBP 5m	N/A	Interest holiday in first 12 months; guarantee fee waived, lenders pay a fee; loan terms set by each lender	Up to 6 years	21.3	0.8%
United Kingdom (Bounce Back Loan Scheme, BBLS)	SME	100%; GBP 2’000–50’000 but maximum of 25% of turnover	N/A	no fees, interest or repayment of principal in the first 12 months; after 12 months: interest rate of 2.5%	Up to 6 years	42.4	1.6%
USA (Paycheck Protection Program, PPP - CARES Act)	SME	100% to end-2020; up to the lesser of USD 10m or a payroll-based amount	30 Jun 2020 (extended to 8 Aug 2020)	1% interest rate; optional interest payment holiday for first 6 months	2 (5) years	477.8	2.5%

*Source*: Baudino (2020) and national sources

## Data Availability

The datasets used and/or analyzed during the current study are available from the corresponding author upon reasonable request and only with permission of the Swiss National Bank, the State Secretariat for Economic Affairs, the Federal Statistical Office, the Institute of Financial Services of the Lucerne University of Applied Sciences and Arts and the Faculty of Business and Economics of the University of Basel, as restrictions apply to the availability of these data and so are not publicly available. Declarations

## Abbreviations

AUD: Australian dollar
BBLS: Bounce Back Loan Scheme
BUR: Betriebs- und Unternehmensregister (registry of commerce)
CAD: Canadian dollar
CARES Act: Coronavirus Aid Relief and Economic Security Act
CBILS: Coronavirus Business Interruption Loan Scheme
CEBA: Canada Emergency Business Account
CHF: Swiss franc
CRF: COVID-19 refinancing facility
EUR: Euro
FINMA: Eidgenössische Finanzmarktaufsicht (Swiss financial market supervisory authority);
FOPH: Federal Office of Public Health
FSO: Federal Statistical Office
FTE: Full-time equivalent
GBP: Pound sterling
GDP: Gross domestic product
HKD: Hong Kong dollar
KfW: Kreditanstalt für Wiederaufbau (Credit Institute for Reconstruction)
NOGA: Nomenclature Générale des Activités économiques (General Classification of Economic Activities)
OECD: Organisation for Economic Co-operation and Development
ONET: Occupational Information Network
PPP: Paycheck Protection Program
SECO: Secrétariat d’Etat à l’économie (Swiss State Secretariat for Economic Affairs)
SFGS: Special Financing Guarantee Scheme
SME: Small and medium-sized enterprises
SNB: Swiss National Bank
UID: Unique identifier
UK: United Kingdom
US: United States
USD: US dollar
VAT: Value-added tax
ZHAW: Zürcher Hochschule für Angewandte Wissenschaften (Zurich University of Applied Sciences)

## Footnotes

1 These are the conditions as originally announced and effective until the end of the application period.

2 In that sense, we focus on the extensive margin and not the intensive margin of the programme. We focus on the former, as we can better separate demand and supply effects for the participation decision, as explained in Section 3.

3 In the literature, there are various definitions of zombie firms. We had to resort to a definition, which can actually be operationalised based on the data we have available. In particular, we only have a cross-section of group-wise (headcounts within sectors) indicators of indebtedness and profitability. The lack of a time-series dimension, for instance, excludes the possibility to apply the OECD-definition of a zombie firm, which is as follows: “Zombie firms are defined as firms aged ≥10 years and with an interest coverage ratio <1 over three consecutive years.” (see, e.g. OECD Economic Policy Paper No. 21, December 2017: “Confronting the zombies: policies for productivity revival”).

4 In general, participation could result from a precautionary motive, where the financing is not actually needed to make current payments but just held for precautionary reasons. However, the trigger for that precautionary behaviour should still be the determining factors explained in the main text.

5 The respondents were taken from an online pool of *LINK*, which provides a nationwide, representative sample of 115,000 individuals.

6 In the first half of April 2020, 205 managers of Swiss companies were asked about their business situation and their response to the pandemic.

7 Public credit guarantee programmes have existed at least since the beginning of the twentieth century. According to Green (2003), more than 2000 such schemes existed in almost 100 countries.

8 Seminal contributions in this literature are, for example, Bernanke and Gertler (1989), Bernanke, Gertler, and Gilchrist (1999), Carlstrom and Fuerst (1997), Holmström and Tirole (1997) and Kiyotaki and Moore (1997).

9 See, for example, Honohan (2010). Offering a public loan guarantee programme can be undertaken for other reasons as well, for instance, to kick-start a lending process by allowing participants to gain experience, which might be important for the economic development of a particular sector, region or country.

10 Beck et al. (2010) survey 76 credit guarantee schemes in 46 developed and developing countries.

11 Apart from the federal COVID-19 loan programme, there have been loan support programmes set up by individual cantons and joint programmes, such as the one offered for start-ups. Quantitatively, the federal programme is by far the largest programme.

12 There are approximately 300 firms (out of a total of more than 600,000) with an annual turnover of more than CHF 500 million.

13 In case of hardship, the original emergency decree establishing the programme allowed for an extension of another 2 years.

14 For COVID-19 plus loans, each bank can fix its own interest rate for the remaining 15% of the loan. For both tranches, the emergency decree stipulated that the interest rate for the subsequent years would be determined by the government and would reflect market conditions.

15 See Art. 6, COVID-19-Solidarbürgschaftsverordnung.

16 Loan supply in terms of the loan volume, however, is also determined by supply-side factors, such as the parameters of the programme. For instance, the loan volume is capped at 10% of a firm’s revenue. The potential total loan volume hence varies across cantons. This fact is exploited by Kaufmann (2020) in his analysis of the macroeconomic employment effects of the programme.

17 Few COVID-19 standard loan applications were initially rejected. Anecdotal evidence points to the fact that rejections were due to incorrectly filling in the application. Credit risk considerations may be more relevant for COVID-19 plus loans, but rejection rates seem to have been low also in this segment.

18 The CRF was established simultaneously and in coordination with the COVID-19 loan programme. By mid-June, banks drew via the CRF liquidity amounting to almost two-thirds of the entire COVID-19 credit limits.

19 The FINMA temporarily excluded central bank reserves from the leverage ratio calculation.

20 Those firms in that 99% of firms employ two out of three employees in Switzerland. For more information, please visit the following website: https://www.kmu.admin.ch/kmu/en/home.html.

21 The September version of JANUS that we use reflects the loans outstanding as of 31 July 2020 (when the COVID-19 loan programme ended). The BUR reflects Swiss firms as of 17 August 2020.

22 We build sectoral categorisations based on the General Classification of Economic Activities (NOGA) two-digit codes.

23 Unfortunately, the BUR registry does not contain financial information such as cash holdings or leverage. Hence, we resort to group-level financial variables in the analysis (see Table 1). Similarly, we do not have information on non-COVID-19 loans that a firm might have secured. This lack of information is important unless the decision to request a non-COVID-19 or a COVID-19 loan occurs at random, which is most likely not the case for larger firms. Section 5.5 shows that our results are robust when excluding larger firms.

24 During the lockdown, essential sectors (such as food stores, pharmacies, petrol stations, banks or railway stations) were allowed to operate freely. These sectors are assigned an index value of zero by Faber et al. (2020). The index value is also set to zero for workers who report working in the public sector.

25 Both indices capture the lockdown restrictions similarly. The correlation coefficient between the two indices is -0.65 when using household observations for which the indices can be mapped.

26 The SECO short-time work numbers are as of April 2020.

27 CompNet documents individual firms across several countries. The survey consists of a sample of non-financial firms with at least one employee. For Switzerland, the CompNet 2017 sample covers around 20% of the country’s total firm revenue; see CompNet (2020) for further details.

28 These more granular data on firm liquidity are not publicly available.

29 See SECO (2016), *Studie zur Finanzierung der KMU in der Schweiz*.

30 The FSO computed the equity (after profit distribution) to total assets ratio; we recovered the debt ratio by taking the complement, i.e. the residual to 100%. These more granular data on debt/equity are not publicly available.

31 We obtain similar regression results for probit models. As the fitted log-likelihood of the logit model slightly outperforms the probit model, we use logit models for our estimations.

32 Depending on the testing regime, fatalities might be a better measure of actual virus intensity. However, this aspect might be more important in international comparisons, as testing was relatively homogeneous across Switzerland.

33 As indicated in Table 2, this also holds when controlling for lockdown restrictions (see column 5). Hence, it seems that there is a separate effect of virus intensity over and above that of the lockdown.

34 The results are unaffected when classifying firms based on alternative and potentially more demanding percentile combinations, such as 60% and 40%, 70% and 30% as well as 80% and 20% (the results are available upon request). We cannot use smaller tails of the distribution to classify zombies because no firm group has both a debt ratio above the 90% and a profitability measure below the 10%. In other words, the CompNet data for Switzerland does not seem to contain *super* zombie groups. Overall, these results might indicate that zombies are not that common in Switzerland, at least not to the degree that they dominate the headcount-sector groups used in CompNet.

35 For more details, see: https://www.bfs.admin.ch/bfs/en/home/registers/enterprise-register/business-enterprise-register.html.
